# Ultrasound-Assisted Marination: Role of Frequencies and Treatment Time on the Quality of Sodium-Reduced Poultry Meat

**DOI:** 10.3390/foods8100473

**Published:** 2019-10-11

**Authors:** Elena S. Inguglia, Catherine M. Burgess, Joseph P. Kerry, Brijesh K. Tiwari

**Affiliations:** 1 Department of Food Chemistry & Technology, Teagasc Food Research Centre, Ashtown, D15 KN3K Dublin, Ireland; elena.inguglia@teagasc.ie; 2 Food Packaging Group, School of Food and Nutritional Sciences, University College Cork, T12 K8AF Cork, Ireland; joe.kerry@ucc.ie; 3 Department of Food Safety, Teagasc Food Research Centre, Ashtown, D15 KN3K Dublin, Ireland; kaye.burgess@teagasc.ie

**Keywords:** ultrasound, salt, reduction, marination, poultry

## Abstract

The objective of this study was to evaluate the influence of high-power ultrasound (US) to accelerate marination of chicken breast; the effect of ultrasonic frequencies and marination times were investigated on samples containing full sodium levels (FS) or 25% sodium reduction, either by reducing NaCl (R50) or by its partial substitution with KCl (SR). Chicken breasts were marinated in plastic bags immersed in an ultrasonic bath operating with a frequency of 25, 45 or 130 kHz for 1, 3 or 6 h at a temperature of 2.5 ± 0.5 °C. Chicken marinated using US had a significantly higher uptake (*p* < 0.05) of sodium compared to control samples (no US) marinated for the same amount of time. No significant changes were observed in the quality parameters of sonicated chicken samples compared to controls. However, significant decreases (*p* < 0.05) in lipid oxidation were observed in SR samples when treated by US. These results suggest the use of ultrasound in the meat processing industry as a novel technology for enhancing marination processes and formulation of reduced sodium meat products.

## 1. Introduction

The consumer’s interest for marinated chicken in Europe is continually growing, not only due to chicken meat being an important source of high-quality protein, vitamins, and minerals but also due to the increased demand for ready-to-eat convenience foods [1,2,3,4]. Marinade refers to a mix of ingredients, in a dry or liquid form, which are applied to uncooked food to enrich flavour and textural properties. Marinades, which usually contain salt, as well as other ingredients, such as sugar, flavour and colouring agents, diffuse into the meat by an osmotic gradient, from the higher concentration of marinade on the outside, to the lower concentration, i.e., inside the meat [4]. The original marination method of soaking the meat in a liquid solution for 12–24 h is generally time consuming and, prolonged soaking time can lead to enzymatic softening, structural damage or even bloating of the surface. Thus, a variety of marinating systems, including multi-needle injection, massaging, tumbling or vacuum tumbling, are currently used in the food industry to accelerate marinade absorption into meat. When it comes to meat products, texture, colours and lipid oxidation are the most important parameters influencing the quality and acceptability of meat and poultry; lipid oxidation is particularly important in poultry, whose fats are highly unsaturated. It is known that heat treatment can both enhance lipid oxidation as well as negatively affect meat texture; therefore, it is of interest for the food industry to develop new technologies that allow the processing of meat, poultry and derived products in a non-oxidant way. Sodium chloride (NaCl) in processed chicken is generally around 1.2% to 2%, dictating the salt content in a typical marinade to range from 4% to 10%. Addition of NaCl is needed not only to improve the flavour and tenderness of the meat but also for its well-recognized ability to improve the binding properties of poultry meat by increasing the solubility of the myofibrillar proteins [5,6,7]. Despite the important role of salt, the current consumer interest for foods with a reduced amount of sodium, has lead the food industry to look for new ways to reach the required salt reduction without impacting the quality of the foods. In terms of ingredients, various alternative approaches have been used to create low-sodium meats, among which, the most common is the replacement of the sodium with potassium, in the form of potassium chloride (KCl). In terms of processing, high-intensity ultrasound, or ultra-sonication (20–100 kHz), is a promising technology where applications have already been seen; the impact of acoustic waves on the food matrix can generate high pressure and local high temperature through which effects can be seen on mass transfer, meat tenderization, shelf life extension and improved functional properties of emulsified products without effects on other quality properties [8,9,10,11,12,13,14]. Although the positive effects of power ultrasound for mass transfer processes has been demonstrated, very few studies have looked at how different US frequencies can influence marination time and meat qualities, such as texture and secondary lipid oxidation in poultry meat. Although the use of marination methods like immersion and injection can improve meat quality, these methods can still take a long time to achieve the desired attributes [15]. Hence, the aim of this study was to evaluate the use of different ultrasonic frequencies as a tool to accelerate salt diffusion for chicken marination under cold processing conditions; the effects of each frequency (25, 45, 130 kHz) on key meat quality parameters were investigated. Moreover, to investigate the potentiality of US as a tool to help achieve lower sodium targets in processed poultry, quality parameters of US-marinated samples containing up to 25% less sodium were evaluated.

## 2. Materials and Methods

The study was divided into two sections: in the first part, the effects of different ultrasonic frequencies (25, 45 and 130 kHz) and treatment times (1, 3 or 6 h) on the uptake of sodium, cook losses, texture profile and lipid oxidation were investigated. Based on these results, in the second part, selected frequencies and treatment times where used to investigate the changes in the quality parameters of chicken meat, marinated with a 50% salt-reduced formulation, either alone (R50) or with substitution by KCl (1:2, SR).

### 2.1. Sample and Marinade Preparation

Chicken breast fillets, obtained from a local supermarket, were used for all the experiments. The muscles were stored at 4 °C prior to processing. The marination mix was made using distilled water in which 8% (*w*/*v*) NaCl, 4% (*w*/*v*) citric acid, 10% (*w*/*v*) garlic powder, 4% (*w*/*v*) ginger powder, 3% (*w*/*v*) black pepper powder, 4% (*w*/*v*) turmeric powder and 0.01% (*w*/*v*) brown sugar were dissolved and stored at 4 °C before use. For the sodium-reduced mixture, 8% NaCl (FS) was reduced to 4% NaCl (R50) or substituted with 4% NaCl + 4% KCl (*w*/*v*) (SR) in addition to the other ingredients. Each chicken fillet was cut into 3 pieces of ~40 g each and individually sealed with the marinade in a 1:2 (*w*/*w*) ratio between the meat and the liquid.

### 2.2. Ultrasound Treatment

Marination was carried out in ultrasonic bath (US) systems operating at 25, 45 and 130 kHz (Elma Schmidbauer GmbH, Singen, Germany) with a corresponding power of 4.7, 5.5 and 7.2 W, respectively. The energy input (Power, W) was measured using the calorimetric method, outlined previously by [16]. A temperature of 2.5 ± 0.5 °C was maintained constant by an external refrigerator circulator, a heat exchanger and a pump as described previously [11]. Each piece derived from the same chicken breast was individually marinated for 1, 3 or 6 h. Samples were treated six at a time with a random allocation of the bags within the US bath. After marination, the excess liquid was removed, and the samples transferred to a clean bag before further processing. Control samples followed the same preparation, but the marination step was carried out by immersion for 1, 3, 6, 16 or 24 h at 4 °C. All treatments were carried out in four replicates.

### 2.3. Sodium Content

Distilled water from a Milli-Q water purification system (Millipore, Bedford, MA, USA) was used for the preparations of the standard and sample solutions. For sodium determinations (Na^+^) ~10 g of meat was blended for 30 s using a food blender; samples were weighed into porcelain dishes, dried overnight and placed on a Gallenkamp hot plate until completely burnt. Burned samples were placed in a muffle furnace to ash at 525 °C for approximately 8–10 h. Ash was dissolved with a few drops of hydrochloric acid (HCl) and diluted to a volume of 50 mL in a volumetric flask. Lanthanum chloride was dissolved in distilled water and diluted to 0.1% (*w*/*v*) and 1 mL was added to each flask. Sodium was quantified by an Atomic Absorption Spectrometer 3110 (Perkin Elmer, Waltham, MA, USA) using sodium standard solutions and calibration curves to correlate the relative absorbance. Samples were measured in duplicate for each sample.

### 2.4. Cooking Method and Cook Loss

Samples were cooked in a water bath at 75 °C until an internal temperature of 72 °C was reached; final internal end-point temperatures were recorded using a food thermometer (Hanna Instruments, Bedforshire, UK). Cook loss was calculated as the weight change before and after cooking, as described by [17]. 

### 2.5. Formation of Secondary Lipid oxidation Products

Formation of secondary lipid oxidation products on raw and cooked chicken at the end of the marination time was quantified as TBARS (thiobarbituric acid reactive substances) as described previously [18]. Calibration was performed using a standard curve of 1, 1, 3, 3-tetraethoxypropane (TEP—Fisher Scientific Ireland Ltd., Dublin, Ireland). Results were calculated in duplicate from four independent samples and expressed as µg of malondialdehyde (MDA) per g of meat. 

### 2.6. Texture Profile Analysis (TPA)

Force–time deformation curves on cooked chicken samples were obtained using a 35 mm flat circular anvil attached to a 500 N cell on an Instron Universal testing machine (Model No. 5543, Instron, Bucks, UK). In general, a minimum of four cores (1.5 cm diameter, 1.5 cm height) were prepared from every sample. Each core was axially compressed with a double compression cycle up to 60% of the original portion height. Hardness (*N*), the peak force during the first compression cycle, was measured from the graph and the other texture profile parameters calculated as: cohesiveness (dimensionless), the ratio of the area of the first and second compression; springiness, calculated as the recovery (mm) of the sample between compressions and chewiness, calculated as gumminess × springiness.

### 2.7. Statistical Analysis

Average values and standard deviations of replicates (*N* = 4) were determined from the data. Statistical difference between treatment means were compared using analysis of variance (ANOVA) followed by Tukey post hoc test at α < 0.05 level. To measure the strength of the linear relationship between variables, the Pearson correlation coefficient *r*, at a *p* < 0.05 and *p* < 0.01 was estimated for independent and dependent variables; the correlation coefficient is a number between −1 and 1 that indicates the extent to which variables are linearly related. Statistical analyses and correlation analysis were performed using SigmaPlot version 13, from Systat Software, Inc., San Jose, California, CA, USA.

## 3. Results and Discussion

### 3.1. Effects of Ultrasonic Frequencies on Sodium Uptake

Sodium chloride, phosphates and sugars are considered important ingredients of marinades, as they can improve meat tenderness and flavour. Marination also tends to increase the water binding capacity of meats, therefore, reducing cooking losses and improving meat texture [5,7]. A strong significant positive correlation was observed between the treatment time and sodium content (*r* = 0.531, *p* < 0.001) of marinated chicken breast, meaning that the sodium content increased with increasing treatment times; a positive, but moderate interaction (*r* = 0.286, *p* < 0.001) was also observed between the frequencies used and the overall sodium content, suggesting an increase in the sodium content at increased frequency, Table 1. 

Accordingly, chicken breast marinated using ultrasound had a statistically significant higher sodium content (*p* < 0.05) than traditionally marinated chicken, Table 2. A treatment time of 1 h did not show significant difference (*p* > 0.05) between the three frequencies used (25, 45 and 130 kHz), where sodium (Na^+^) reached a similar level of 0.40%; however, sodium uptake in sonicated samples was significantly higher (*p* = 0.001) than the chicken marinated by immersion (<0.20%), Table 2.

**Figure 1:**
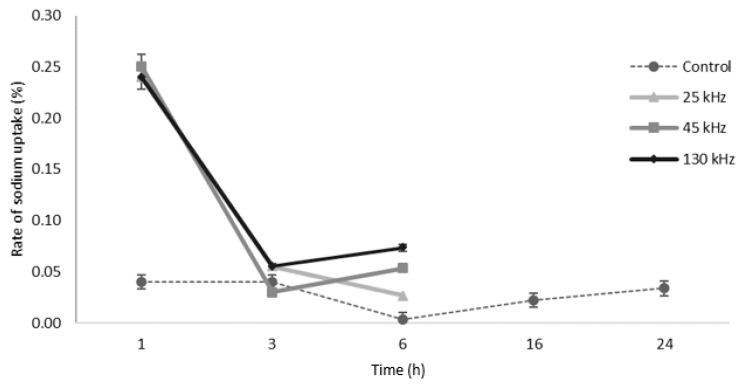
Rate of sodium uptake based on treatment time. Sodium uptake (g/100 g of sample) in chicken samples marinated with 8% NaCl solution in an ultrasonic bath (25, 45, 130 kHz) or by immersion (Control). The rate at each time interval was calculated as ((Na) _T2_ − (Na) _T1_)/(T_2_ − T_1_).

Interestingly, other authors observed a similar salt concentration of 1.18% (estimated sodium ~0.47 g) in beef samples sonicated for one hour at 40 kHz, suggesting that time, more than the frequency used, can influence salt content [10]. Similar to the trend observed for the 1 h treatment, after 3 h of US-assisted marination, no statistically significant differences (*p* > 0.05) were observed between the three ultrasonic frequencies used, where no increase of sodium content compared to the 1 h processing time was observed; however, sodium values were again significantly higher (*p* = 0.001) than chicken samples treated without ultrasound (0.27% Na^+^), Table 2. The same pattern was observed after 6 h of US marination, where the sodium level increased to ~0.60% for ultrasound-treated samples, which was significantly different (*p* = 0.001) from the control. The effect of marination time on sodium content among the different US frequencies, showed a significant difference (*p* = 0.018) only at 130 kHz, where a concentration of 0.68% of sodium was reached after 6 h, versus 0.62% and 0.58% obtained with 25 and 45 kHz, respectively. In chicken pieces marinated without ultrasound, a total of 16 h was needed to reach a similar sodium concentration in the meat (0.50% Na^+^), and up to 24 h (0.77% Na^+^) to reach higher levels than those observed after only 6 h of US-assisted marination. Another study showed the potential use of high-power ultrasound in poultry-processing methods by studying the dye penetration in the meat over 15 or 30 min; using an ultrasonic bath operating at 40 kHz, they observed that ultrasound increased the amount of dye inside the samples by 6% and 13% after 15 and 30 min, respectively [19]. Similarly, a higher sodium level and a more homogenous distribution were reported in beef samples marinated with a bath system operating at 11 W/cm^2^ [10]. These results suggest that ultrasound processing, at the conditions used in this study, has a strong positive effect on salt diffusion in marinated chicken breast, and its effects are strictly correlated to the treatment time rather than the frequency used. However, by measuring the rate of sodium uptake during ultrasound processing, we observed that at all frequencies, the highest rate of sodium uptake (0.24%) was measured only in the first hour of treatment, and follow by a significant decrease at increasing treatment time, Figure 1. For the control, in contrast, the rate of sodium uptake even if significantly lower, was constant over time; therefore, explaining the impact of time over sodium content for standard marination. The mechanisms responsible for the higher diffusion of sodium in the US meat have been hypothesized as due to the improved mass transfer caused by either a mechanical effect of cavitation, leading to the formation of microjets that can promote erosion or pitting of the surface, or damage caused by shock waves; according to this, it is the collapsing of the bubbles inside the liquid which cause the erosion of the solid surface; therefore, improving diffusion mechanisms [20].

Cooking loss values of control and US-marinated samples are also presented in Table 2. No statistically significant differences (*p* > 0.05) were observed between control and chicken samples marinated using ultrasound at 25, 45 or 130 kHz. These results are, however, consistent with other findings, observing no changes in cooking loss values of beef steak treated with high-power ultrasound with a frequency of 40 kHz [21,22]. On the other hand, other studies have shown a reduction in cooking losses with increased treatment time, which had been explained as a result of the increased salt content, or due to the thermal effects of ultrasound in the medium [13,23,24]. In our study, however, no significant differences (*p* > 0.05) were observed for cooking losses when the treatment time for the same frequency was extended from 1 to 6 h. Moreover, no significant correlation between cooking loss and sodium content (*r* = 0.085, *p* = 0.539) was observed, Table 1. Decreased cooking losses after ultrasound treatment, have been generally linked to the denaturation of the meat myofibrillar proteins; in this study, the absence of differences between samples treated with or without ultrasound, may be attributed to the conditions used for ultrasound processing: lower intensities, cold temperatures of the surrounding liquid and having the meat sealed in a polyamide/polyethylene bag, could have had an impact on limiting the water loss and thermal denaturation of the protein structure in the meat.

### 3.2. Texture Profile Analysis

Texture profile analysis (TPA) is a method that can simulate the mechanical process of mastication by measuring the compression force of a probe during two cycles of food deformation [25]. The main advantage of TPA is that multiple parameters can be assessed in one measurement. Generally, for meat products, the parameters measured are hardness, springiness and cohesiveness; the three altogether permit the calculation of chewiness [26]. TPA parameters of control and sonicated samples for 1, 3 and 6 h treatments are presented in Table 3. 

No significant differences (*F* = 0.97, *p* = 0.495) between US-treated and untreated samples were observed for hardness, which represents the amount of force necessary to achieve deformation in the sample. Based on previously reported studies, modification of meat hardness due to sonication processes can only be achieved at higher ultrasonic intensities and for longer processing times [8,22,27]. As observed for cooking losses, the absence of protein denaturation due to the chosen treatment conditions could explain the absence of tenderizing effects on the meat. Cohesiveness is a parameter that describes how well a food retains its form between the two compressions; a significant negative correlation (*r* = −0.563, *p* < 0.001) was measured between cohesiveness and hardness but no significant differences were observed between samples treated at different US frequencies compared to control. This data suggests that the structure of the meat tissue remains almost intact, especially if short ultrasonic treatments are employed [8]. Springiness, or how well a product physically springs back after it has been deformed during the first compression, and chewiness, did not show significant differences (*p* > 0.05) between the marination methods.

### 3.3. Formation of Secondary Lipid Oxidation Products

It is known that the use of ultrasound in an aqueous medium induces the formation of strong oxidizing agents such as OH radicals, H_2_O_2_ and ozone, which can further initiate or enhance other oxidative reactions [28]. The lipids present in food are highly susceptible to oxidative reactions and, moreover, the addition of salt can favour further lipid oxidation, causing undesirable changes in flavour and decreasing meat quality [28,29]. The possible mechanisms of sodium chloride as a pro-oxidant have been attributed to the ability of NaCl to disrupt cell membrane integrity, therefore, facilitating the access of oxidizing agents to lipid substrates. The results of TBARS analysis for the marinated chicken samples are presented in Table 4. Statistically significant effects (*p* < 0.05) were observed on the oxidative status of marinated chicken samples treated with a frequency of 25 kHz compared to the control when measured as cooked meat. However, no significant differences were observed (*p* > 0.05) between the raw samples marinated with or without ultrasound and at all treatment times. Similarly, other studies have also shown that ultrasound did not cause any significant increase in the oxidation of fats in cured beef samples [30,31]. In addition, it is possible that no changes were detected due to the natural low-fat content of the marinated chicken meat.

### 3.4. Quality Parameters of Reduced-Salt Marinated Chicken

Based on the results of the frequencies and treatment times tested for US-assisted marination, and the respective quality parameters discussed above, a selected frequency of 130 kHz and a treatment time of 1 h, were chosen to look at the effects of ultrasound on chicken immersed in a marinade with a 1:2 replacement of NaCl with KCl (SR) or with only half of the original salt content (R50, 4% *w*/*w*). Potassium additives, such as KCl, confer a similar salty taste and are, therefore, one of the most often used replacements for sodium in sodium-reduced food products [12,32]. When looking solely at molecular weights of sodium chloride (58.44) and potassium chloride (74.55), 27.5% more potassium chloride must be used to provide the same molar concentrations; however, previous research has shown positive results using equal replacement on a weight basis [33]. Sodium content and cook losses of chicken breast marinated using both solutions are shown in Table 5. 

**Figure 2:**
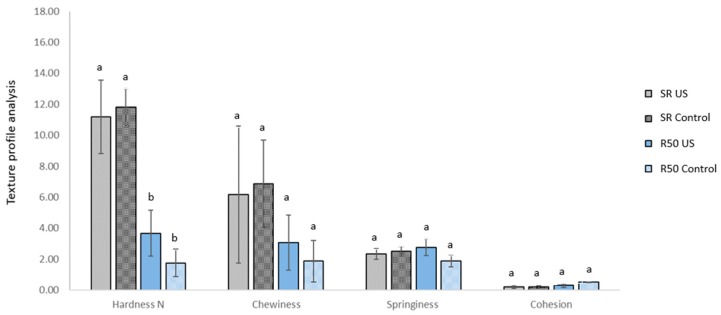
Texture Profile Analysis (TPA) of samples marinated with a 1:2 substitution of NaCl with KCl (SR) or with a 50% reduction of NaCl (R50). Chicken samples were marinated with a frequency of 130 kHz for 1 h. Values are compared with a chicken sample marinated without ultrasound for 16 h (Control). Values represents means ± standard deviation (SD, *N* = 4). Different letters represent significant differences at *p* < 0.05 for each attribute.

As expected from the previous experiment, no differences (*p* > 0.05) were found between the total sodium content of samples treated with or without ultrasound at the selected treatment times; samples marinated with US reached the target of a 25% sodium reduction levels in 1 h compared to the 16 h needed for the control treatment. The achieved sodium reduction is in line with the general labelling legislation, regulation (EC) 1924/2006, stating that to claim that sodium/salt has been reduced, the reduction in content has to be at least 25% compared to a similar product [34]. Partial replacement of sodium chloride with potassium chloride (SR) did not show any significant effect (*p* > 0.05) on the cooking loss, when compared to samples marinated with NaCl only (R50), Table 5. A similar conclusion was presented by [35], where replacement of sodium chloride by potassium chloride by up to 50% in marinated rabbit meat, did not change the technological traits such as pH, colour, texture, cooking loss and yield. According to the authors, as normally only the Cl^-^ anions are responsible for the swelling of myofilaments, substitution with Na^+^ and K^+^ cations did not modify the effects of chloride on meat quality parameters [35,36]. Comparison of the texture profile analysis of samples from the two sodium-reduced marinades is presented in Figure 2. 

**Figure 3:**
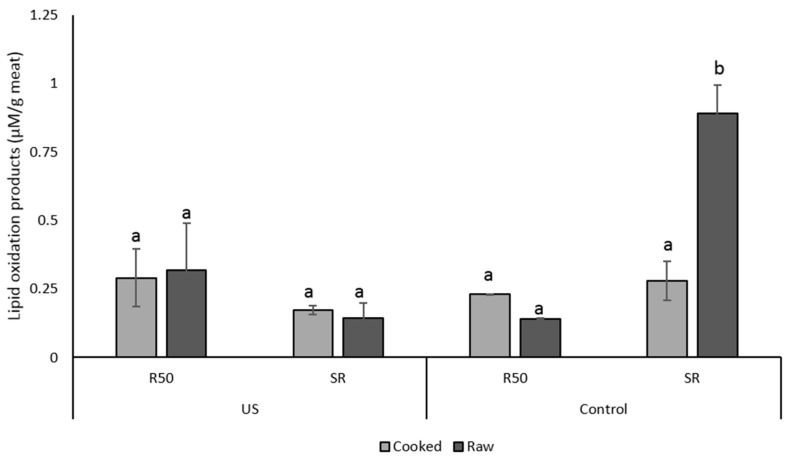
Formation of secondary lipids oxidation products in chicken marinated with NaCl/KCl (SR) or reduced-salt marinade (R50); Chicken samples were marinated with a US frequency of 130 kHz for 1 h. Values are compared with chicken samples marinated without ultrasound for 16 h (Control). Values represents means ± standard deviation (SD, *N* = 4). Different letters are significant at *p* < 0.05.

Significant differences (*p* < 0.05) were observed between the two formulations; chicken marinated with a 50% reduction of salt, had a significantly lower hardness (<4 N) compared to samples marinated with the sodium replacer (>11 N); value not significantly different from the full-sodium chicken samples 13.17 ± 1.26 N, Table 3. Salt reduction alone, without the addition of KCl to compensate for the functional properties of NaCl, negatively impacted the texture of the product. Other studies observed that substitution of NaCl with KCl (10%–60%) did not affect the texture of dry-cured loin and, similarly, cooked ham formulated with a 50% replacement of NaCl with KCl was found to provide better binding and acceptable sensory scores, while hams formulated with 70% NaCl/30% KCl or 30% MgCl_2_ were not found to be different in terms of flavour, tenderness and acceptability compared to hams made with 100% salt [37,38,39]. The use of ultrasound, however, was not shown to have a significant impact on the texture of the marinated chicken when compared to the control. Changes between samples were, however, observed on the oxidative status of fatty acids. A statistically significant (*p* = 0.001) higher level of fatty acid oxidation was observed on raw chicken samples marinated with the sodium replacer KCl for 16 h, when compared to samples processed by ultrasound, Figure 3. 

However, no significant differences were observed between the samples processed in the reduced-salt marinade. On cooked chicken meat, no differences were observed between sonicated samples compared to the controls, as well as between chicken marinated with or without KCl. Ultrasound treatment showed no negative effects on the oxidative stability of fatty acids but instead, at the frequency, temperature and treatment time used for marination, processing with ultrasound seemed to improve the oxidative stability of the fatty acids in a chicken sample marinated with the sodium replacer, KCl.

## 4. Conclusions

Ultrasound significantly decreases the time needed for the marinade to penetrate the meat compared to a standard marination technique. No differences where observed between the US frequencies tested; however, higher uptake of sodium was achieved by applying 130 kHz, reaching the sodium targets in only 1 h, compared to 16 h needed for the traditional immersion method. No changes were observed in the texture and lipid oxidation profile of the US-marinated chicken samples with standard sodium content. However, at lower sodium levels and without the replacement of sodium with other mineral salts, ultrasound processing could not replace the functional role of salt, resulting in significant changes in the meat texture. Nonetheless, when NaCl was substituted with KCl and treated by ultrasound, a statistically significant decrease in the fatty acid oxidation was observed, while the other quality parameters remained unaltered compared to full-sodium products. These findings suggest that ultrasound technology offers the potential to improve and speed up existing marination processes; further studies on the role of ultrasound in combination with sodium-replacing ingredients could provide the food industry with new processing options to achieve sodium reduction in processed poultry meat. 

## Figures and Tables

**Table 1:** Pearson’s correlation coefficients between the treatment variables and meat quality parameters ^a^.

	Time	Frequency	CL	Na	Hd	Cw.	Sp.	Ch.	MDA-c
Frequency	0.399 **	-	-	-	-	-	-	-	-
CL	0.394 **	0.199	-	-	-	-	-	-	-
Na	0.531 **	0.286 **	0.085	-	-	-	-	-	-
Hd.	0.102	−0.045	−0.035	−0.026	-	-	-	-	-
Cw.	−0.131	0.316	−0.062	−0.415 **	0.164	-	-	-	-
Sp.	0.018	0.299	0.084	−0.338 **	−0.132	0.611 **	-	-	-
Ch.	−0.177	0.212 **	0.018	−0.199	−0.563 **	0.513 **	0.273	-	-
MDA-c	−0.112	−0.445 **	−0.018	0.093	0.054	−0.239	−0.157	−0.134	-
MDA-r	0.356 **	0.309 **	0.558 **	0.037	−0.164	0.072	0.139	0.144	−0.088

^a^ Frequency used; treatment time (time); cooking losses (CL); sodium content (Na); hardness (Hd); chewiness (Cw); springiness (Sp.); cohesiveness (Ch), malondialdehyde (MDA) (µg/g of meat) cooked sample (MDA-c) and MDA on raw sample (MDA-r). Values are significant at ** *p* < 0.001.

**Table 2:** **Sodium uptake and cook losses:** Effects of ultrasonic frequencies (25, 45, 130 kHz) on the mineral uptake (g/100 g of meat) and cook loss of chicken breast marinated for 1, 3 and 6 h. Values for sodium uptake for chicken sample marinated without ultrasound (Control) after 16 and 24 h are also presented. Values represents means ± standard deviation (SD, *N* = 4).

	Sodium (%)				Cook Loss (%)			
Time	Control	25 kHz	45 kHz	130 kHz	Control	25 kHz	45 kHz	130 kHz
1 h	0.19 ± 0.03 ^aB^	0.39 ± 0.13 ^aA^	0.40 ± 0.06 ^aA^	0.46 ± 0.11 ^aA^	20.46 ± 2.12 ^aA^	20.42 ± 3.52 ^aA^	18.20 ± 4.08 ^aA^	22.49 ± 4.25 ^aA^
3 h	0.27 ± 0.07 ^bB^	0.50 ± 0.07 ^aA^	0.46 ± 0.06 ^aA^	0.47 ± 0.08 ^aA^	19.74 ± 6.14 ^aA^	18.67 ± 2.17 ^aA^	19.06 ± 1.79 ^aA^	16.91 ± 5.30 ^aA^
6 h	0.27 ± 0.04 ^bC^	0.58 ± 0.08 ^aB^	0.62 ± 0.04 ^aB^	0.68 ± 0.09 ^bA^	19.74 ± 1.97 ^aAB^	22.89 ± 3.58 ^aAB^	23.62 ± 2.30 ^aAB^	16.52 ± 4.69 ^aB^
16 h	0.50 ± 0.11 ^c^				47.32 ± 7.11 ^b^			
24 h	0.77 ± 0.14 ^d^				21.33 ± 1.49 ^a^			

Different letters in the same column (^abc^) or in the same row (^ABC^) indicate values significantly different at *p* < 0.05.

**Table 3:** Texture Profile Analysis (TPA) ^1^ parameters; Chicken breast samples were marinated using power ultrasound operating at a frequency of 25, 45 or 130 kHz for 1, 3 or 6 h. Values for chicken samples marinated without ultrasound (CNT) are also presented. Values represent means ± standard deviation (SD, *N* = 4).

	FS Marination (8% NaCl)					
		1 h	3 h	6 h	16 h	24 h
Hd	CNT	13.90 ± 2.89 ^a^	11.83 ± 1.12 ^a^	14.47 ± 5.29 ^a^	13.17 ± 1.26 ^a^	14.27 ± 4.50 ^a^
	25	10.89 ± 2.89 ^a^	16.35 ± 3.36 ^a^	12.56 ± 4.40 ^a^	-	-
	45	13.45 ± 4.44 ^a^	15.62 ± 5.02 ^a^	15.78 ± 1.32 ^a^	-	-
	130	7.64 ± 5.44 ^a^	13.83 ± 8.81 ^a^	13.15 ± 7.08 ^a^	-	-
Cw	CNT	10.61 ± 1.75 ^a^	7.14 ± 4.17 ^a^	8.21 ± 5.29 ^a^	6.33 ± 1.14 ^a^	4.73 ± 1.72 ^a^
	25	5.06 ± 1.74 ^a^	3.65 ± 1.13 ^a^	6.21 ± 2.29 ^a^	-	-
	45	7.88 ± 3.39 ^a^	4.78 ± 1.54 ^a^	3.58 ± 1.10 ^a^	-	-
	130	5.21 ± 3.59 ^a^	3.75 ± 3.12 ^a^	6.33 ± 2.44 ^a^	-	-
Sp	CNT	2.60 ± 0.21 ^a^	2.45 ± 0.37 ^a^	2.42 ± 0.15 ^a^	2.43 ± 0.17 ^a^	2.28 ± 0.39 ^a^
	25	2.29 ± 0.10 ^a^	2.06 ± 0.19 ^a^	2.16 ± 0.16 ^a^	-	-
	45	2.49 ± 0.19 ^a^	2.15 ± 0.12 ^a^	2.01 ± 0.16 ^a^	-	-
	130	2.17 ± 0.78 ^a^	2.02 ± 0.40 ^a^	2.23 ± 0.19 ^a^	-	-
Ch	CNT	0.30 ± 0.10 ^a^	0.23 ± 0.08 ^a^	0.22 ± 0.06 ^a^	0.20 ± 0.02 ^a^	0.16 ± 0.08 ^a^
	25	0.20 ± 0.07 ^a^	0.10 ± 0.03 ^a^	0.26 ± 0.20 ^a^	-	-
	45	0.24 ± 0.06 ^a^	0.16 ± 0.05 ^a^	0.10 ± 0.04 ^b^	-	-
	130	0.38 ± 0.10 ^a^	0.16 ± 0.07 ^a^	0.25 ± 0.13 ^a^	-	-

^1^ Hardness (Hd, N); Chewiness (Cw, J); Springiness (Sp, m); Cohesiveness (Ch). Values followed by a different letter in the same column for each parameter are significantly different, at *p* < 0.05.

**Table 4:** TBARS values; Chicken breast samples were marinated using power ultrasound operating with a frequency of 25, 45 or 130 kHz for 1, 3 or 6 h. Values for a chicken sample marinated without ultrasound (CNT) are also presented. Values represent means ± standard deviation (SD, *N* = 4). TBARS values are expressed as µg MDA/g of meat on cooked and raw products.

	FS Marination (8% NaCl)					
		1 h	3 h	6 h	16 h	24 h
**Cooked samples**	**CNT**	0.12 ± 0.03 ^aB^	0.17 ± 0.09 ^aA^	0.13 ± 0.06 ^aA^	0.21 ± 0.01 ^a^	0.19 ± 0.03 ^a^
	**25**	0.46 ± 0.28 ^aA^	0.43 ± 0.30 ^aA^	0.29 ± 0.19 ^aA^	-	-
	**45**	0.16 ± 0.08 ^aAB^	0.47 ± 0.29 ^aA^	0.26 ± 0.09 ^aA^	-	-
	**130**	0.21 ± 0.07 ^aAB^	0.20 ± 0.11 ^aA^	0.26 ± 0.04 ^aA^	-	-
**Raw samples**	**CNT**	0.09 ± 0.03 ^aA^	0.26 ± 0.12 ^aA^	0.22 ± 0.11 ^aA^	0.57 ± 0.33 ^a^	0.22 ± 0.03 ^a^
	**25**	0.19 ± 0.04 ^aA^	0.12±0.06 ^aA^	0.17±0.12 ^aA^	-	-
	**45**	0.17±0.06 ^aA^	0.16±0.02 ^aA^	0.20±0.03 ^aA^	-	-
	**130**	0.26±0.20 ^aA^	0.27±0.17 ^aA^	0.33±0.15 ^aA^	-	-

Values followed by different letters in the same row (^abc^) or in the same column (^ABC^) for each treatment are significantly different, at *p* < 0.05.

**Table 5:** Mineral content and cook loss of sodium-reduced chicken; effects of US processing (130 kHz) for 1 h on the sodium uptake (g/100 g of meat) of chicken breast marinated with a 1:2 substitution of NaCl with KCl (SR) or only with half of the original salt content (R50). Values are compared to samples marinated without ultrasound (Control) for 16 h. Values represents means ± standard deviation (SD, *N* = 4). Values followed by different letters in the same column are significantly different, at *p* < 0.05.

	Sodium (%)		Cook Loss (%)	
	SR	R50	SR	R50
Control (16 h)	0.31 ± 0.05 ^a^	0.19 ± 0.04 ^a^	20.72 ± 0.96 ^a^	21.88 ± 7.65 ^a^
130 kHz (1 h)	0.25 ± 0.02 ^a^	0.22 ± 0.03 ^a^	21.61 ± 1.87 ^a^	20.31 ± 0.96 ^a^
